# Phenotypic responses to temperature in the ciliate *Tetrahymena thermophila*

**DOI:** 10.1002/ece3.6486

**Published:** 2020-07-01

**Authors:** Vanessa Weber de Melo, Robert Lowe, Paul J. Hurd, Owen L. Petchey

**Affiliations:** ^1^ Department of Evolutionary Biology and Environmental Studies University of Zurich Zurich Switzerland; ^2^ The Blizard Institute Queen Mary University of London London UK; ^3^ School of Biological and Chemical Sciences Queen Mary University of London London UK

**Keywords:** adaptation, experimental evolution, morphology, phenotypic plasticity, Temperature, *Tetrahymena*

## Abstract

Understanding the effects of temperature on ecological and evolutionary processes is crucial for generating future climate adaptation scenarios. Using experimental evolution, we evolved the model ciliate *Tetrahymena thermophila* in an initially novel high temperature environment for more than 35 generations, closely monitoring population dynamics and morphological changes. We observed initially long lag phases in the high temperature environment that over about 26 generations reduced to no lag phase, a strong reduction in cell size and modifications in cell shape at high temperature. When exposing the adapted populations to their original temperature, most phenotypic traits returned to the observed levels in the ancestral populations, indicating phenotypic plasticity is an important component of this species thermal stress response. However, persistent changes in cell size were detected, indicating possible costs related to the adaptation process. Exploring the molecular basis of thermal adaptation will help clarify the mechanisms driving these phenotypic responses.

## Graphical abstract

Using experimental evolution, we evolved a model ciliate in a novel high‐temperature environment for more than 35 generations. We observed long lag phases, a strong reduction in cell size, and modifications in cell shape. Adapted populations exposed to their original temperature displayed plastic responses in many traits, indicating phenotypic plasticity is an important component of this species thermal stress response.

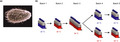

## 1 INTRODUCTION

Temperature is one of the most important abiotic factors, influencing all levels of biological organization, from cell function to ecosystem dynamics (Johnston & Bennett, 1996). Understanding how organisms respond and adapt to a novel temperature has, therefore, been the focus of multiple studies exploring physiological, ecological, and evolutionary mechanisms of temperature response (Angilletta, 2009; Clarke, 2003). The current climate change crisis revived the interest in this research field, since understanding how populations will respond to new temperatures is of fundamental importance (Hoffmann & Sgrò, 2011; Walther et al., 2002).

Microorganisms, like many other ectotherms, are particularly sensitive to the temperature of their environment as it directly affects their metabolism and many physiological processes (Pörtner et al., 2006). Since microorganisms play key functions in all ecosystems, understanding their responses to temperature is essential to forecasting the future of ecosystems (Singh, Bardgett, Smith, & Reay, 2010). Besides their ecological importance, many of these organisms have short life cycles, large population sizes and are readily manipulated in the laboratory, offering many possibilities to experimentally study thermal adaptation over multiple generations (Elena & Lenski, 2003; McDonald, 2019).

The effect of temperature on the size of microorganisms is one of the most studied morphological responses to temperature. Most species display smaller cell sizes when grown at higher temperatures, a response known as the temperature–size rule (Atkinson, 1994). Besides cell size, many important phenotypic traits such as cell shape (Trueba, van Spronsen, Traas, & Woldringh, 1982) and swimming behavior are also affected by temperature (Beveridge, Petchey, & Humphries, 2010; Schneider & Doetsch, 1977).

Organisms can use different mechanisms to survive in a novel temperature. Many species display phenotypic plasticity, that is, the same genotype can generate multiple phenotypes, in response to a change in their environment (see review Murren et al., 2015). Plastic responses can be further separated into two different types, developmental plasticity, that is, traits that vary according to the environment during development, but are then irreversible during an organism's life spam; or traits that are context‐dependent and show variation in the same individual, such as behavior or metabolic reactions, sometimes called phenotypic flexibility (Piersma & Drent, 2003). Acclimation, that is the adjustment of physiological traits to environmental conditions, is one example of phenotypic flexibility (Piersma & Drent, 2003; Wilson & Franklin, 2002). Populations can also adapt to a novel temperature, which occurs when genetic changes lead to a population with higher fitness in the new environment. Temperature adaptation can lead to the evolution of specialization and have costs to an organism, such as reduced performance in the ancestral or other environments (Huey & Kingsolver, 1989). These costs are predicted by theory and have been observed in previous experiments (Bennett & Lenski, 2007; Jin & Agustí, 2018). Phenotypic plasticity and adaptation are not mutually exclusive mechanisms; in fact it, is likely that they are combined in many responses to environmental change (Davis & Shaw, 2001; Gienapp, Teplitsky, Alho, Mills, & Merilä, 2008).

Several studies have experimentally examined thermal adaptation in microorganisms such as bacteria (Hall, Neuhauser, & Cotner, 2008; Sandberg et al., 2014; Tenaillon et al., 2012; Trueba et al., 1982), phytoplankton (Padfield, Yvon‐Durocher, Buckling, Jennings, & Yvon‐Durocher, 2016; Schlüter et al., 2014), and yeast (Caspeta et al., 2014; Huang, Mei‐Yeh, Chang, & Li, 2018), but this topic has been little explored in protists. To better understand temperature adaptation in this group, we chose the ciliate protist *Tetrahymena thermophila* as our model system. This is one of the best‐studied species of protists, and it is able to grow in a wide range of temperatures (Laakso, Löytynoja, & Kaitala, 2003).

Different studies have estimated the mutation rate of this species, which is an important feature for experimental evolution studies. *T. thermophila* has two nuclei, the micronucleus (MIC), which is diploid and has a germline function during sexual reproduction, and the macronucleus (MAC), which is polyploid (~45 N) and transcriptionally active. Brito, Guilherme, Soares, and Gordo (2010) performed a mutation accumulation experiment and found high mutation rates in the MAC of this species, estimated at *U* = 0.0333 per haploid macronucleus genome per generation. Long, Paixao, Azevedo, and Zufall (2013), in a second mutation accumulation experiment, estimated a MIC mutation rate of *U* = 0.0047 (95% credible interval: 0.0015, 0.0125) per haploid MIC genome per generation and found no evidence that the mutation rate of the MAC is different than the MIC. This estimate is lower than in the previous experiment by Brito et al., but in the range of other eukaryotes. Together with large population sizes, *T. thermophila* is therefore a suitable model for experimental evolution.

**FIGURE 1:**
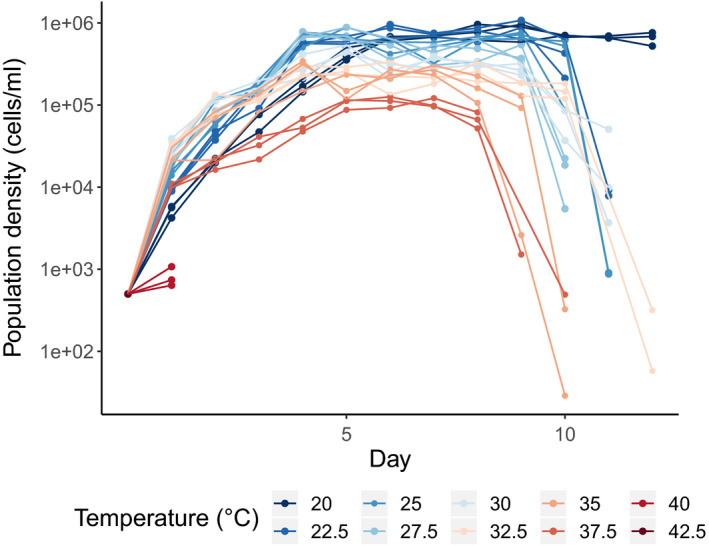
Population dynamics of *T. thermophila* strain 1630/1U growing in 10 different temperatures. Each line represents one replicate population, and the colors indicate the temperature in which the population was grown

In this study, we monitored the population dynamics and cell morphology of four replicate populations of *T. thermophila* exposed to 38°C, a highly stressful condition. With this, we tested whether populations can survive in a temperature near lethality without previous acclimation. Different mechanism can be involved in this process, from phenotypic plasticity to adaptation to the new environment. When exposed to such a high temperature, we predict the growth rate of *T. thermophila* populations will have an immediate and strong decrease. This prediction is based on the observed reductions of population growth at temperatures above 37.5°C (see Figure 1, which contains results from a pilot experiment described in the Methods section), and also because this temperature is close to the thermal limit of the species (Laakso et al., 2003). Long‐term exposure to 38°C will likely result in growth rates similar to the ones in nonstressful temperatures, if populations are able to adapt to the novel temperature.

We hypothesize a short‐term negative effect of temperature on cell size, as predicted by the temperature–size rule (Atkinson, 1994) and established for many protists (Atkinson, Ciotti, & Montagnes, 2003). The shape of protist cells can be affected by stressful environmental conditions (Dias, Mortara, & Lima, 2003; Kovács, Hegyesi, Köhidai, Nemes, & Csaba, 1999), by the presence of predators (Hammill, Petchey, & Anholt, 2010; Kuhlmann & Heckmann, 1985) and is related to dispersal behavior (Pennekamp, Mitchell, Chaine, & Schtickzelle, 2014). Clear hypotheses about how and why cell shape would change with temperature are currently absent. Nevertheless, we present the effects of temperature on cell shape, since exploratory analyses may yield insights into this understudied trait.

After many generations at this novel temperature, the populations were then returned to the control temperature, a benign environment. This enabled us to test if survival at high temperatures had any costs to the organisms. If there are costs associated to survival at high temperature, we expect to see reduced growth rates when adapted populations return to the control condition. Similar patterns should also be observed for the morphological traits, with reduced cell sizes when populations return to the control condition, since larger cells have a higher fitness. Opposite results would indicate the evolution of generalists or the presence of a plastic response.

## 2 MATERIALS AND METHODS

### 2.1 Strain and culture conditions

**FIGURE 2:**
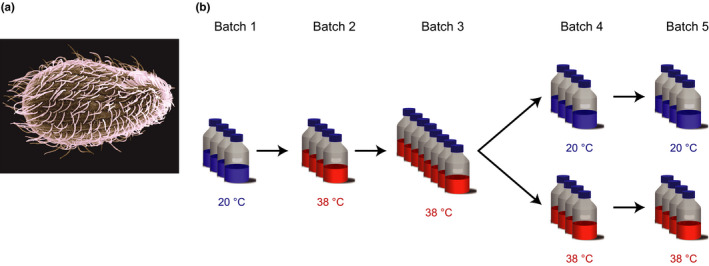
Image of the ciliate *Tetrahymena thermophila* (a) and experimental design of the temperature experiment (b). In the schematic of the experiment, each bottle represents one replicate batch culture, and the colors indicate the temperature in which the culture was grown. (a) Image credit: Dr. Aaron J. Bell

All experiments were performed with the ciliate *T. thermophila* (Figure 2a) strain 1630/1U cultured in axenic conditions in 2% proteose peptone medium. This strain was acquired from the Culture Collection of Algae and Protozoa and grown during many generations at 15°C to acclimatize it to our laboratory conditions. We did not initiate the stock cultures from single cells; this strain likely contains very low genetic variability due to a long history of serial transfers (Ketola, Laakso, Kaitala, & Airaksinen, 2004). *T. thermophila* only reproduced clonally in all experiments, since a single strain with one mating type was used.

The medium used in all experiments was prepared with proteose peptone from the same manufacturer batch, ensuring homogeneous conditions across all experimental replicates. The bottles and the medium used in the experiments were sterilized in an autoclave, and all sampling procedures were performed in sterile conditions. Microbial contamination was regularly checked during the experiments by plating a sample of each culture on an agar plate incubated at 37°C for 24 hr. To ensure our treatments were reliable, we monitored every 15 min the actual temperature in the incubators used to grow the experimental populations. In all incubators, the mean daily temperature presented a standard deviation smaller than 0.28°C.

### 2.2 Temperature range of *T. thermophila*

We performed a pilot experiment to identify the temperature range in which this *T. thermophila* strain is able to grow, exploring ten different temperatures from 20 to 42.5°C, in intervals of 2.5°C. Three replicate populations were grown in each temperature for a period of 13 days. The initial population density of all replicates was 500 cells/ml, and the populations were monitored daily with videos to measure population density.

We observed cell division in all tested temperatures except at 42.5°C (Figure 1). When grown in temperatures between 20 and 37.5°C, populations immediately entered exponential growth and reached high and stable carrying capacities. At 40°C, populations initially increased in density but collapsed after two days, indicating this temperature is close to the upper thermal limit of this strain.

To explore temperature adaptation in this species, we chose 20°C as the control temperature of the experiment and 38°C as the adaptation temperature. At 20°C, populations grow very well and reach carrying capacity in a short period of time, while 38°C is a stressful temperature that creates a strong selective pressure.

### 2.3 Experimental design

The temperature adaptation experiment was performed with populations of *T. thermophila* growing in axenic batch cultures in 2‐L bottles with 500 ml of medium. The cultures were placed in incubators with controlled temperature, no light and in shakers to increase aeration. The experiment was initiated with four replicate populations from the same stock culture, giving rise to four separate evolving lineages. There were five consecutive batch cultures of each lineage that lasted a total of 41 days (Figure 2b).

All the batch cultures started at a low density (500 cells/ml), and once they reached carrying capacity, new batch cultures were started from a small aliquot of the previous batch. The volume of this transferred aliquot depended on the population density of the culture at the end of the previous batch, to ensure that all new batch cultures started with the same cell density (500 cells/ml). Each batch had a different duration, since the time to reach carrying capacity changed during the experiment, in large part due to the temperature treatment. The shortest batch lasted five days, while the longest batch lasted 12 days.

In the first batch, the four replicate cultures were grown at 20°C, and in the second batch, the four replicates started the adaptation to 38°C. Each culture in the second batch originated two cultures in the third batch, resulting in eight cultures still growing at 38°C. In the fourth batch, one of each paired culture was moved back to 20°C, while the other culture remained at 38°C. In batch five, the cultures continued in the temperature experienced in the previous batch.

Population sizes in this experiment were large, ranging from 374,000 cells at the beginning of each batch culture, when population density was 500 cells/ml, reaching population sizes of 187 × 10^6^ at 38°C and 433 × 10^6^ at 20°C. Using the MAC mutation rate estimated by Brito et al. (2010), we expect 2,997 × 10^3^ mutations in the shortest batch at 38°C (8 generations), which is a significant proportion of the genome of this species, considering a MAC genome size of 104 Mbp. Hence, we believe the duration of the experiment sufficient to allow adaptation to occur.

### 2.4 Video monitoring and processing

The cultures were monitored daily after the second or the third day of each batch, and every day in batch 5. These minor differences in the monitoring schedule compensated for minor differences in the timing of population dynamics. Monitoring included estimation of population abundances and multiple morphological measurements. On the monitoring days, each culture was sampled twice, since duplicate assessments provide more accurate estimates of the population abundance. Each sample consisted of 1 ml of culture. The samples were placed in counting chambers, and the videos were taken on a stereomicroscope (Leica M205 C) mounted with a digital CMOS camera (Hamamatsu Orca C11440, Hamamatsu Photonics, Japan) with 1.57× magnification. When population density was high, samples were diluted with fresh medium before taking the videos. Each video comprised 125 frames in 5 s and monitored 40.26 μl of sample. The videos were processed using the R package BEMOVI version 1.0 (Pennekamp, Schtickzelle, & Petchey, 2015), which extracts morphological information of all the moving cells in the field of view.

### 2.5 Data analysis

In total, 94,344 cells were measured, with an average of 513 ± 26.9 cells monitored per population per day. The number of detected cells was used to estimate population density throughout the experiment. We calculated the minimum number of generations (*G*) that took place in each batch culture with the equation *G* = ln(*A*_max_/*A*_0_)/ln(2), where *A*_0_ is the minimum population abundance, and *A*_max_ is the maximum population abundance.

All statistical analyses were performed using R (R Core Team, 2019). We used a Gompertz model (Zwietering, Jongenburger, Rombouts, & Klaas van't Riet, 1990) implemented in the R package growthrates version 0.8.1 (Petzoldt, 2019) to estimate the maximum growth rate and the duration of the lag phase in each population per batch. The model fittings can be seen in Figure 4a. Despite different optimization strategies, this model presented low *R* ^2^ values for population 3 in batch 4 at 38°C, indicating it could not properly model the growth dynamics of this population. We therefore calculated the maximum growth rate of this population as the log_10_ difference in abundance between the maximum and the minimum population abundance, divided by the time period between these days. The lag phase of this population was set to zero, since it quickly entered exponential growth.

We used two morphological measurements in this study, the cell area and the cell shape (the ratio between the longer and the shorter axes of the cell). The morphological measurements were averaged per population per day, and the coefficient of variation among individuals for cell size was calculated per population per day. We recorded information related to movement behavior, such as swimming speed, but did not include these data in the present study. We acquired videos at room temperature, which differed from the growing temperature, and some samples required dilution due to high density. Therefore, we were not confident that movement behavior recorded from the videos would reflect effects of the temperature treatment of the experiment.

To investigate the effect of temperature on population dynamics and cell morphology, we performed two separate analyses, first on the populations at 38°C and then on the populations that returned to the control temperature. We analyzed six different traits, the population growth rate, the lag phase, the mean cell size, its coefficients of variation and its variance, and the mean cell shape. For each of these six response variables, the average change relative to the control (batch 1) was calculated for each population in each batch. Relative change in the morphological traits was expressed as percent, and absolute differences were used for the lag phase and growth rate. We used the package MCMCglmm version 2.29 (Hadfield, 2010) to fit linear mixed effects models to each of the six response variables, modeling the effect of temperature across all batches in each variable. All models included population lineage as a random effect and time as a fixed effect, and we used relatively uninformative priors (*V* = 1, nu = 0.002). We ran each model for 2,000,000 iterations, with a burn in of 30,000 iterations and storing every 1,000th iteration. We assessed model convergence with autocorrelation analyses and with trace plots using the package coda version 0.19‐3 (Plummer, Best, Cowles, & Vines, 2006).

## 3 RESULTS

### 3.1 Population dynamics

**FIGURE 3:**
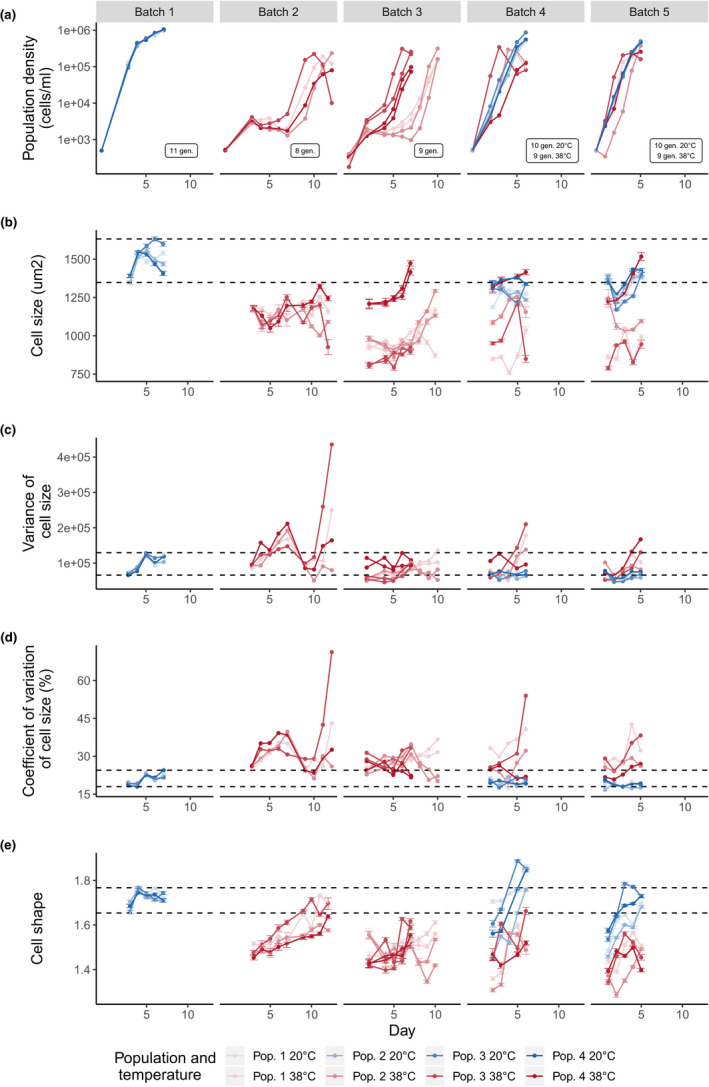
Population dynamics and morphological traits of each *T. thermophila* population during the temperature experiment. Population density (a), mean cell size (b), variance of cell size (c), coefficient of variation of cell size (d), and mean cell shape (e) are shown for each population and for each batch separately. Minimum number of generations that took place in each batch is shown in boxes in plot (a). Error bars indicate standard errors of means for cell size and cell shape (b and e). The colors indicate the temperature in which the population was grown, and the shade represents the population replicate. Dashed lines mark the range of observed values at the control temperature (20°C) in the first batch of the experiment

**FIGURE 4:**
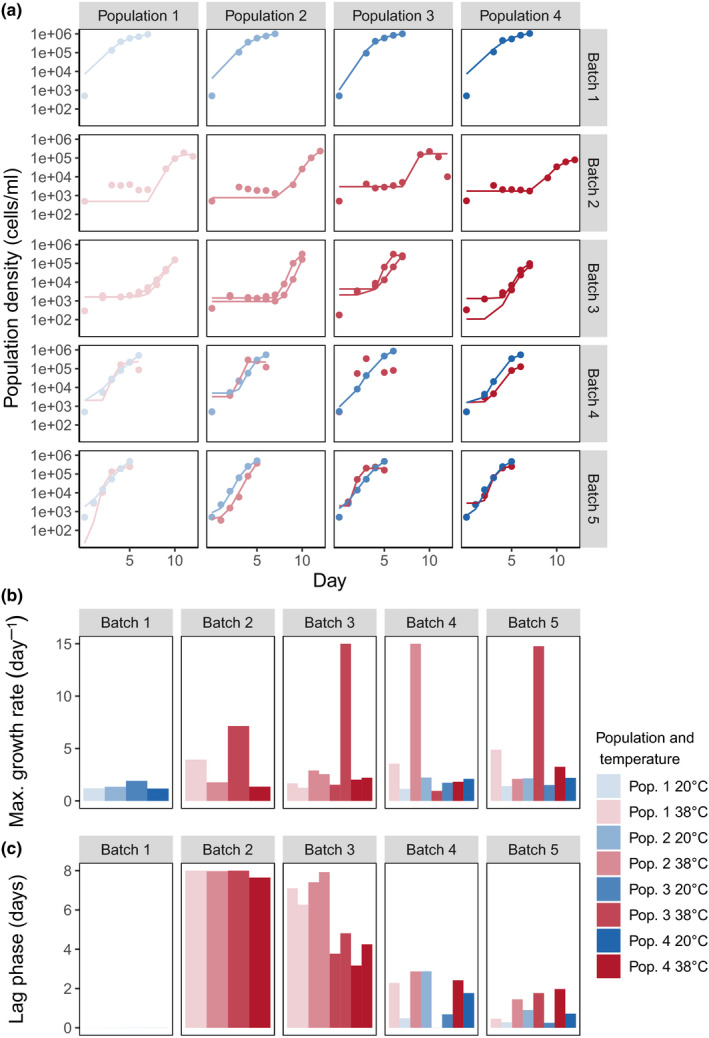
Growth dynamics of *T. thermophila* during the temperature experiment. Gompertz model was used to estimate demographic parameters. (a) Points show population abundances, and lines show the fitting of the model. No model fit is shown for population 3 in batch 4 at 38°C since growth parameters were manually calculated (see Section 2). Maximum growth rate (b) and lag phase (c) of each *T. thermophila* population per batch culture. In all plots, the colors indicate the temperature in which the population was grown, and the shades represent the population replicate. The lag phases in batch 1 are estimated as smaller than 1 × 10^−5^ and are not visible in the plot

All replicate populations grew well in the control temperature of 20°C in the first batch (Figure 3a), immediately entering exponential phase and reaching carrying capacity in a few days. When populations started to grow at 38°C, they entered a long lag phase and exponential growth started only after 7 days (Figure 4b,c). Similar dynamics were observed in batch 3, even though a few populations displayed a shorter lag phase. In batch 4, the populations that continued at 38°C displayed a much reduced lag phase, similar to the populations that went back to the control temperature. Batch 5 had similar population dynamics to what was observed in the previous batch. The four batches in which *T. thermophila* populations experienced 38°C comprise a minimum of 35 generations.

**FIGURE 5:**
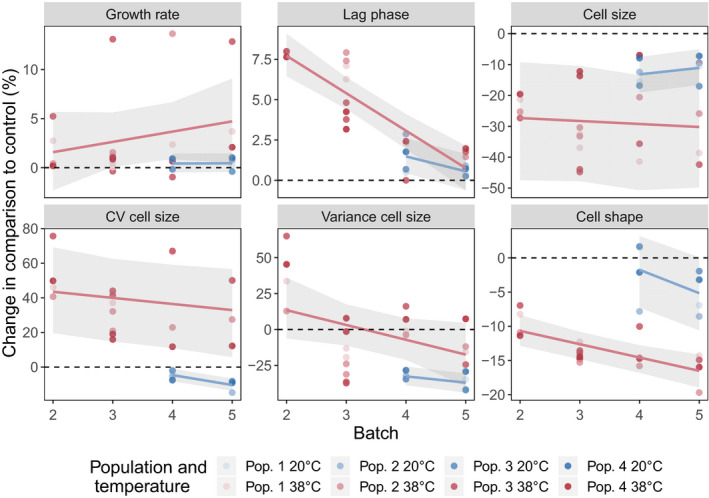
Change in population dynamics and morphological traits of *T. thermophila* populations. Change in maximum growth rate, lag phase, cell size, variance of cell size, coefficient of variation (CV) of cell size and cell shape are shown, for each population and for each batch separately, using the control cultures at 20°C in batch 1 as a reference. Maximum growth rate difference is expressed in day^−1^, and lag phase difference is expressed in days, while all other values are expressed as percent difference. The lines represent the fitted mixed effects models and the shaded areas represent the 95% credible interval (see Section 2 for details). The colors indicate the temperature in which the population was grown, and the shades represent the population replicate. The dashed lines mark no change in comparison to the control

Temperature strongly affected the lag phase of *T. thermophila* cultures. In the first batch at 38°C, lag phase was increased by 7.7 days [6.3; 8.9] (here and later in square brackets is the estimated 95% credible interval) in comparison to the control temperature, but it gradually decreased during the experiment. In the final batch, lag phase at 38°C was not different from the lag phase at the control temperature (0.7 days [−0.6; 2.1]; Figures 4c and 5). Maximum growth rate slightly increased during the experiment, and we found a significant effect of temperature, with an increase of 4.5 [0.4; 8.7] at the end of batch 5 (Figures 4b and 5). Maximum population density decreased at 38°C, but it remained lower throughout all batches (Figure 3a). In batch 4, the populations that moved back to 20°C displayed an immediate increase in maximum population density, similar to the maximum density observed in batch 1.

We observed a marked variation in population dynamics across the population replicates at 38°C, especially in batches 2 and 3, while population dynamics at 20°C were much more similar. Some populations exhibited a much larger maximum growth rate in specific batches (Figure 4b), and there was also significant variation in lag phase duration, for example, in batch 3, in which populations 1 and 2 presented a lag phase two times longer than populations 3 and 4 (Figure 4c).

### 3.2 Cell morphology

Temperature had a strong and long‐lasting effect on cell size and shape. There was an immediate decrease of 27.3% [9.3; 47.5] in cell area when populations moved to the higher temperature (Figures 3b and 5). This size reduction was maintained throughout the entire experiment and populations displayed a cell area reduction of 30.2% [11.2; 49.8] at the final batch. When populations moved back to 20°C after many generations growing at 38°C, cell size recovered and the mean cell area increased, but populations did not return to the initial conditions observed in batch 1 (−11.1%, [−16.6; −5.0]).

To explore the variation in cell sizes during the experiment, we modeled both the variance and the coefficient of variation (CV) of cell size in response to temperature, which showed slightly different patterns, since the CV is scaled by the sample mean. The CV of cell area significantly increased at 38°C through the entire experiment (32.9% [5.75; 56.6] in batch 5; Figures 3d and 5), while the variance in cell size was not different than the control (−17.4% [−39.5; 4.79] in batch 5; Figures 3c and 5). Taken together, they indicate a slightly wider range of cell sizes when populations first moved to the novel temperature, but a reduction in variation toward the end of the experiment (Figure 5).

Similar patterns were observed in cell shape, as can be seen in the mean cell shape of the populations during the adaptation experiment (Figures 3e and 5). Cells became rounder as they adapted to 38°C (16.5% [14.1; 19.0] rounder in batch 5), and cell shape returned to more elongated formats when populations moved back to 20°C (only 5.2% [0.1; 10.6] rounder in batch 5).

## 4 DISCUSSION

The results of this experiment clarify phenotypic and population responses of *T. thermophila* exposed to a high temperature environment for more than 35 generations. Immediate effects of high temperature included a large increase in the lag phase. There was, however, no effect on the growth rate of the populations. The long lag phases in the first batch at 38°C returned to control levels after around 26 generations, while growth rate at 38°C slightly increased during the experiment. These results provide further evidence to the importance of lag phase in microbial population dynamics (Bertrand, 2019) and its importance during responses to environmental change.

Phenotypic effects were observed in the morphology of *T. thermophila*, with a prevalence of smaller and rounder cells at high temperature. Previous experiments using *Tetrahymena* species have also observed reductions in cell size at high temperatures (DeLong et al., 2017; James & Read, 1957), a pattern also present in other ciliates (e.g., Weisse, Stadler, Lindström, Kimmance, & Montagnes, 2002), but the duration of these experiments was much shorter, comprising only a small number of generations. Our study shows that cell size is immediately reduced at high temperatures and remains lower as populations evolve. Despite this general trend, one of the replicate populations displayed a much smaller cell size reduction (population 4, Figure 3b), indicating that larger cells are also a viable phenotype at high temperatures. Furthermore, cell size showed an increased variation at the high temperature environment, while at 20°C cells had a more uniform morphology. Stressful conditions often lead to a higher phenotype variability (Hoffmann & Hercus, 2000), but this pattern is another indication that more than one phenotype is viable in this environmental condition.

Different hypotheses try to clarify the mechanisms through which temperature affects body size. Although most of them were developed to explain the plastic response of the temperature–size rule, these hypotheses are also applicable for long‐term adaptive responses to temperature. One hypothesis relates smaller cells at high temperatures to higher metabolic rates and therefore higher oxygen demands. Since oxygen diffusion is reduced as temperature increases, a reduction in cell size compensates for that (Atkinson, Morley, & Hughes, 2006). Another possible explanation is based on growth rate being more affected by temperature than development rate, which would lead to organisms dividing at a younger age and thus being smaller (Zuo, Moses, West, Hou, & Brown, 2012). A third mechanism is based on body size optimization between the organisms demand and the expected resource supply in a given temperature (DeLong, 2012). These hypotheses are not mutually exclusive, and all are relevant to our study system. Each of these hypotheses received support from theoretical models and experimental data, but their importance for the observed patterns is still under debate.

Little is known about the effect of temperature on cell shape in *T. thermophila* and in other ciliates. DeLong et al. (2017) grew *T. thermophila* in three different temperatures, 20, 26, and 32°C, and although variation in cell shape was observed, no clear pattern related to temperature was found. A few studies exposed populations of *T. thermophila* to different stressful conditions and have also observed rounder cells (Dias et al., 2003; Nilsson, 2005). Taken together, these experiments indicate that round cells could be related to harsh environmental conditions in general, and not only to high temperature. The rounder cells could also be connected to malfunction of cytoskeleton proteins at high temperature, since these proteins have an important role in maintaining cell shape (Williams, 2004). Investigations of gene and protein functions in high temperature environments would help clarify these mechanisms.

Possible costs related to thermal adaptation were estimated by analyzing the performance of the populations that returned to the control temperature after more than 18 generations at 38°C. No significant reduction was observed at the growth rate in batches 4 and 5, and cell shape returned to the control levels, indicating little costs related to the high temperature adaptation. Cell size, however, remained smaller even after many generations back in the control temperature (Figure 5), and populations displayed a small increase in lag phase during batch 4 at 20°C (Figure 5). Cell size is an important trait for the fitness of unicellular organisms (Monds et al., 2014) including *T. thermophila* (Long & Zufall, 2015), and the observed pattern may indicate the occurrence of costs when populations adapt to a new temperature. Evidence for costs related to thermal adaptation has been described in previous experiments investigating different microorganisms. Bennett and Lenski (2007) found fitness trade‐offs in *E. coli* populations adapted to 20°C in comparison to the ancestral populations adapted to 40°C. Baker et al. (2018) described trade‐offs in growth rate during adaption to supra‐optimal temperatures in a dinoflagellate, and Duncan, Fellous, Quillery, and Kaltz (2011), using another ciliate, *Paramecium caudatum*, observed trade‐offs when populations were adapted to a specific temperature and became specialists. The dynamics of cell size found in this study indicate that costs may also take place in our study system, but longer experiments are needed to confirm the relevance of this, since trade‐offs might be transient and only present while populations are still adapting to the new environment.

Phenotypic plasticity can also play an important role in thermal adaptation, as observed in experiments with bacteria (Shi & Xia, 2003) and zooplankton (Yampolsky, Schaer, & Ebert, 2013). A plastic response likely also explains some of the patterns in this study, for example, the immediate recover of cell shape when populations return to 20°C in batches 4 and 5 (Figure 5). The long lag phases observed in the batch 2 (Figure 4c), the first batch exposed to the novel temperature, probably also represents a plastic response in the form of acclimation to the new environment. However, populations also displayed longer lag phases in batch 3, and acclimation is not sufficient to explain this pattern, since populations had already been exposed to this novel temperature for multiple generations. Developmental plasticity and adaptive responses are possible mechanisms generating the observed temperature response. Analysis of the molecular basis of this response could clarify the role of plasticity and would also help understanding the mechanisms behind the phenotypic changes during thermal adaptation. Further studies comparing the phenotypic plasticity in the ancestral and in the evolved lineages would also be important to advance our understanding of the relative roles of phenotypic plasticity and genetic variation during temperature adaptation.

One important feature of our study, caused by time and resource constraints, was the use of the ancestral populations grown at 20°C in batch 1 as the control (Figure 2), instead of maintaining populations at 20°C during the entire experiment, or keeping individuals from batch 1 in suspended animation (which is technically difficult for *T. thermophila*) to compare with individuals from later batches in a common garden setting. When comparing the ancestral populations in batch 1 (20°C) and the evolved populations in batch 5 (20°C), as we did, two things differ: 1) prior exposure to 38°C (our treatment) and amount of time in the experimental conditions (i.e., batch). Although we cannot rule out the possibility, we find it unlikely that the amount of time in experimental conditions could account for the observed differences because (a) individuals in batch 1 had already experienced many generations in conditions similar to the experimental conditions; (b) low variation in population dynamics and morphological traits, relative to treatment effects; (c) comparison with previous 20°C populations from the pilot experiment (not shown).

It would be interesting and relevant to investigate if different *T. thermophila* strains display growth and morphological responses to temperature similar to the ones we observed in this experiment. A previous study in the ciliate *Euplotes vannus* observed significant variation in the growth rate thermal dependence across four strains of this species (Walton, Gates, Kloos, & Fisher, 1995), and in *T. thermophila* there is extensive variation of dispersal propensity across genotypes (Pennekamp et al., 2014). Patterns of temperature adaptation in natural populations of *T. thermophila* or other ciliates have received little attention so far. Krenek, Petzoldt, and Berendonk (2012) explored this question in *Paramecium caudatum* populations sampled in a latitudinal range across Europe, but found no indication of local adaptation in this species, while Gächter and Weisse found support for local adaptation to temperature studying the freshwater ciliate *Meseres corlissi* (Gächter & Weisse, 2006). Our study only investigates a single strain of *T. thermophila*, but the results provide evidence that generalists are present in this species as well, since adaptation to a higher temperature had little effect on growth at the ancestral temperature. Studying natural populations of this species would be an interesting comparison to the results obtained in these laboratory experiments and would help better understand the process of thermal adaptation in microorganisms.

## CONFLICT OF INTERESTS

The authors declare no competing interests.

## AUTHOR CONTRIBUTION

**Vanessa Weber de Melo:** Conceptualization (equal); Data curation (equal); Formal analysis (lead); Investigation (equal); Methodology (equal); Project administration (equal); Software (lead); Validation (equal); Visualization (equal); Writing‐original draft (lead); Writing‐review & editing (lead). **Robert Lowe:** Conceptualization (equal); Methodology (equal); Software (supporting). **Paul J. Hurd:** Conceptualization (equal); Methodology (equal). **Owen L. Petchey:** Conceptualization (equal); Formal analysis (supporting); Funding acquisition (equal); Methodology (equal); Resources (equal); Supervision (equal); Writing‐original draft (supporting); Writing‐review & editing (supporting).

## Data Availability

Population abundances and morphological trait data are available on Dryad Digital Repository (https://doi.org/10.5061/dryad.v15dv41tb). Code for all figures and statistical analyses is accessible at Github (https://github.com/vanessawmelo/Temperature_response_Tetrahymena) with the identifier (https://doi.org/10.5281/zenodo.3871397).
